# The Demands of Performance Generating Systems on Executive Functions: Effects and Mediating Processes

**DOI:** 10.3389/fpsyg.2020.01894

**Published:** 2020-07-29

**Authors:** Pil Hansen, Emma A. Climie, Robert J. Oxoby

**Affiliations:** ^1^School of Creative and Performing Arts, University of Calgary, Calgary, AB, Canada; ^2^School and Applied Child Psychology, Werklund School of Education, University of Calgary, Calgary, AB, Canada; ^3^Department of Economics, University of Calgary, Calgary, AB, Canada

**Keywords:** executive functions, inhibition, problem-solving, cognitive flexibility, improvisation, dance, music, theatre

## Abstract

Performance generating systems (PGS) are rule- and task-based approaches to improvisation on stage in theater, dance, and music. These systems require performers to draw on predefined source materials (texts, scores, memories) while working on complex tasks within limiting rules. An interdisciplinary research team at a large Western Canadian University hypothesized that learning to sustain this praxis over the duration of a performance places high demands on executive functions; demands that may improve the performers’ executive abilities. These performers need to continuously *shift attention* while remaining responsive to embodied and environmental stimuli in the present, they are required to *inhibit* automated responses and impulses using the rules of the system, and they strive toward addressing multitasking challenges with *fluidity* and *flexibility*. This study set out to test the mentioned hypothesis deductively and identify mediating processes inductively, using mixed empirical methods. In a small sample experiment with a control group (28 participants; 15 in intervention group, 13 in control group), standardized quantitative tests of executive functions (D-KEFS) were administered before and after an 8-week intervention. Participant-reported qualitative observations from the praxis were also collected throughout the intervention for grounded analysis. Within the limitations of small sample data, we found both statistically significant and trending effects on inhibition, problem-solving initiation, fluidity, and cognitive flexibility. Examining the mediating process, we found that participants experienced significant challenges sustaining the practice halfway through the intervention. The participant-reported solutions to these challenges, which emerged as the strongest behavioral patterns when coding the qualitative data to saturation, were strategies of problem-solving and of re-directing attention. These strategies support and advance our understanding of the effects measured in the standardized tests. In terms of application, our results identify characteristics of PGS that could potentially maintain and strengthen executive functions over and above less demanding performing arts interventions. The results also deliver new insight into how PGS works, which may contribute to the development and teaching of this artistic practice.

## Introduction

If improvisation praxis within performance generating systems (PGS) in theater, dance, and music places high demands on the performers’ executive functions, then might such demands result in positive effects on executive abilities? PGS require performers to continuously *shift attention* and remain responsive to embodied and environmental stimuli in the present, *inhibit* automated responses and impulses using the rules of the system, and strive toward addressing multitasking challenges with *fluidity* and *flexibility*. The specific characteristics of these cognitive demands led our team to anticipate and test quantitative effects on the associated executive functions. In addition, qualitative data was collected and analyzed to further our understanding of the mediating processes that caused measured effects and identify associated challenges and benefits reported subjectively by study participants.

### Theoretical Framework

Executive functioning (EF) is a broad term used to refer to higher cognitive processes that allow one to mediate one’s behavior in response to an ever-changing environment. EF refers to the ability to plan, organize, and maintain an appropriate action to reach a desired goal (Ozonoff et al., 1991), and it is used as an umbrella term to describe higher mental processes. Executive functions encompass the control, supervisory, and/or self-regulatory functions that organize and direct all cognitive activities, emotional responses, and overt behaviors (Isquith et al., 2005). These processes are controlled, rather than automatic, and include the regulation of attention and motor responses, delay of gratification, planning, problem-solving, inhibition of prepotent (or automatic) responses, concept formation, abstract thinking, cognitive and behavioral flexibility, inhibitory control, self-monitoring, working memory, and attention (Carlson et al., 2004; Wiebe et al., 2008).

The term “executive functions” was introduced in relation to the work of Luria (1966) who proposed a cognitive system in charge of intentionality and formulation of thoughts and actions, the identification of goal-appropriate cognitive routines, and evaluation of outcomes. EFs have been shown to be mainly regulated by the prefrontal cortex (PFC) through imaging and neuropsychological studies, though the PFC is not exclusively responsible for these cognitive processes (Goldman-Rakic, 1987; Elliott, 2003; Godefroy, 2003; Rubia et al., 2003). This area of the brain is thought to act primarily as a “control center” that mediates higher-level cognitive functions (Miller and Cohen, 2001). Typically, a quantitative understanding of an individual’s EF abilities is captured using standardized neuropsychological tests that target the PFC, such as the Delis–Kaplan Executive Function System (D-KEFS; Delis et al., 2001).

A variety of overarching skill areas have been found to encompass the cognitive processes of EF. Although there are differing views on exactly what these dimensions are, one prominent perspective incorporates three important skills: inhibitory control, working memory, and cognitive flexibility (Diamond et al., 2007). Two of these EFs, inhibition and cognitive flexibility, are the focus of the current study.

Inhibition involves the ability to refrain from responding with an incorrect, yet prepotent response (Nigg, 2000; Carlson and Moses, 2001; Hala et al., 2003). For example, when completing a stroop task, participants are shown color names written in ink of a different color (i.e., the word “blue” would be written in yellow ink). Participants are asked to identify the color of the ink (i.e., “yellow”), thus requiring them to inhibit the more automatic response of reading the word (i.e., “blue”). Inhibition has been linked to positive social development (Pérez-Edgar et al., 2011), problem-solving (Passolunghi and Siegel, 2001), and academic achievement (e.g., St. Clair-Thompson and Gathercole, 2006).

Cognitive flexibility, also referred to as set-shifting or attentional flexibility, is another key component of EF. Cognitive flexibility is the ability to flexibly switch between rules, where one must disengage attention from one source or rule in order to engage with another (Stahl and Pry, 2005). It incorporates problem-solving, or the ability to work through new tasks applying previous knowledge (Zelazo et al., 2003). For example, in a classic shifting task, participants may be asked to sort a variety of cards based on a specific dimension (e.g., color), to which they receive corrective feedback. During the task, at an unspecified time, the sorting rule changes as indicated by negative feedback. Accordingly, participants must apply their flexible thinking skills and sort the cards using the new rule (e.g., size). To be successful, participants must inhibit previously learned mental sets. Failure to shift results in perseverative errors (i.e., continuing to respond according to the previous set of rules; Anderson, 2002). Poor cognitive flexibility has been linked to difficulties with academics, particularly in mathematics (St. Clair-Thompson and Gathercole, 2006), as well as reduced levels of self-awareness (Moore and Malinowski, 2009).

Research on the role of EFs has begun to integrate into the performing arts, with a focus on the impacts of EF on performing arts interventions. Although the literature in this area is limited, some benefits of music, drama, and dance on EFs have been found. For example, Thaut et al. (2009) utilized therapeutic music training to improve the EFs of patients with traumatic brain injury. This training included a focus on shifting between musical tones, matching rhythmic patterns, and using song as a mnemonic device to aid with recall. Specifically, improvement in cognitive flexibility and attention were found after participating in the intervention program. Comparable effects were found in a study by Biasutti and Mangiacotti (2018). They did a broad test of EF effects of a simple music improvisation intervention (described as imitation-based vocal and rhythm tasks without mention of multi-tasking or set-shifting) for older adults with cognitive impairments. While this study found no results on a test designed to measure cognitive flexibility, global results were found on a verbal fluency test which includes inhibition and shifting between word categories. More conclusive effects were measured on a short test of attention, memory, and visio-spatial perception, which are executive functions that reflect the demands of the intervention more directly. Additionally, Karakelle (2009) examined the impact of a 10-week course that provided students with the opportunity to build improvisation skills through drama, which encouraged students to construct ideas and emotions through symbols, acting, and semi-structured interactions. It was found that divergent thinking, fluency, and cognitive flexibility were improved in a group of postgraduate students following participation in this course (Karakelle, 2009). Finally, Coubard et al. (2011) explored the connection between contemporary dance improvisation and cognitive flexibility in older adults (ages 65–88 years). They found that those who participated in dance improvisation, including free movement creation, stretching, body positioning, practice, and performance, improved in their cognitive flexibility and shifting abilities over those who participated in a more structured course (e.g., Tai Chi or fall prevention program).

A recent meta-analysis (Meng et al., 2019) highlights the impacts of dance intervention on global cognitive abilities as well as executive function in older adults. In this meta-analysis, it was noted that dance did not significantly impact EFs. The types of dance interventions included in reviewed studies of EFs were typically structured (e.g., ballroom dancing) or set (memorized choreography). The one exception mentioned, Coubard et al.’s (2011) study, used a dance improvisation intervention and found an effect.^1^

Looking across these examples, it becomes clear that the discipline of performing arts intervention may be variable, as improvements in EF have been found through music, dance, and drama programs. A similar observation can cautiously be made regarding population groups as effects have been found in studies with younger adults and both healthy and cognitively impaired older adults. What seems to be of greater importance is the focus of the performing arts intervention, as the rehearsal and repetition of memorized material (e.g., ballroom dance steps) does not specifically impact the key EFs of cognitive flexibility, inhibition, and problem-solving. Rather, there is support for the idea that interventions with a greater and more complex improvisation requirement or a specific task-based goal have a closer link to key EFs.

The independent variable of our study, PGS, is a form of improvisation with specified parameters that is comparable across theater, dance, and music. The concept of PGS was first defined by Pil Hansen in 2014 and refers to the generating characteristics of stage works that are not set and repeatable compositions, but rather systematic forms of task-based improvisation, constrained by predefined rules and source materials (Hansen, 2014).^2^ For example, in the theatrical “Lie-line” (first developed by the United Kingdom-based theater company Forced Entertainment and later adapted by the Canadian company Theatre Replacement), actors each select an ‘enhanced’ autobiographical anecdote and take turns sharing their stories over rounds with rules of engagement that gradually evolve from listening, through borrowing and incorporating components from one another’s stories, to interrupting each other. This basic system generates collective stories through self-organizing dynamics of competition, adaptation of memories, and, eventually, disintegration of story and language. The concept of DST draws on dynamical systems theory to define the boundaries (performance context), energy resources (source materials), parameters (tasks and rules), variables (performer training and memories), and attractors (e.g., learning curve, competition, fatigue) of these systems (Hansen, 2015). Analytical and dramaturgical work invested in understanding how these systems generate performance has led to theories about the cognitive demands they likely place on performers (Hansen and House, 2015; Hansen and Oxoby, 2017). In this regard, there is a relevant difference between PGS and the classical forms of improvisation introduced as interventions in the EF studies previously mentioned. Classical approaches to improvisation in the performing arts, such as Paxton’s contact improvisation, Spolin’s theater sports, and Stockhausen’s intuitive music, require performers to “get out of their heads” and instead draw on instinctive and implicit responses while remaining hyperattentive to their co-performers’ shifting responses in the present (Paxton, 1975; Novack, 1986; Hogg, 2011; Drinko, 2013). In other words, performers likely rely on rather than inhibit prepotent (automatic) responses, while avoiding conscious thought processes associated with problem-solving and deliberate set-shifting. New and less commonly used approaches to improvisation are challenging this exclusive focus on the present by articulating the layers of memory and cultural training that inform improvised impulses while performing (Drinko, 2013; Sarco-Thomas, 2014; Midgelow, 2015; Hansen, 2018).

Like these culturally and dramaturgically aware improvisors, creators of PGS are critical of an over-emphasis on presence in classical improvisation methods because the techniques used tend to separate thinking from the body. Their response differs, though: they devise system rules that keep performers consciously and intellectually engaged throughout embodied work. Negative system rules, such as Deborah Hay’s “avoid sequencing” or Ame Henderson’s “don’t copy,” require dancers to consciously inhibit a trained, automated tendency. In these cases, they are the tendencies to string movement into sequences and to entrain to other dancers, arriving at unison and alignment through empathetic copying (Sofianidis et al., 2014; Waterhouse et al., 2014). To work with such rules, performers must render conscious otherwise implicit response habits and then inhibit them (Hansen and Bläsing, 2017, pp. 16–23; Stevens, 2017, pp. 56–57). The tasks created for the systems are often impossible to execute as posed and require that each performer addresses them like problems. When, for example, dancers are tasked to “move continuously” for 60 min but “never repeat movement” by Henderson, they are in fact tasked to remember and consult a vast amount of movement in ways that are humanly impossible. They address this problem by developing strategies that counter their trained tendency toward repetition and provide more limited options (e.g., inhabiting a new space with each movement or shifting relationships with co-dancers or between body parts). As they do so, complex ideas become meaningful and a vast amount of information becomes clustered to avoid working memory overload (Hansen and Oxoby, 2017). As these examples indicate, PGS require performers to both:

(1) Respond in the performance presence with the fluency and attention-shifting that also is common for improvisation techniques, and
(2) Work consciously with shifting rules, inhibition of automated responses, problem-solving, and strategy.

When these observations are considered together, it is reasonable to hypothesize that the EF demands of PGS are significantly higher than the demands of performing memorized material and could be considerably higher than the demands of classical improvisation practices.

## Materials and Methods

### Study Design

The study design applied mixed methods and included a control group. The three following data sets were collected: (1) surveys of demographic variables (completed by all participants), (2) quantitative pre- and post-tests of executive functions (completed by all participants, except one in the intervention study group), and (3) self-reported written observations prepared by participants during the intervention (only completed by the intervention group). Administration and processing procedures will be described later.

The first data set was used to consider variables, the second set was processed statistically to account for intervention effects on executive functions, and the third set was coded thematically to uncover patterns and factors that explain the measured effects. Finally, analytical comparisons and transfer were cautiously made across the quantitative and qualitative findings, which are included in the discussion section of this article.

Participants in the intervention group were recruited by a research assistant from a pool of senior students who were enrolled in a course for degree credit on PGS in the School of Creative and Performing Arts, University of Calgary.^3^ Participants in the control group were recruited from the full cohort of graduate and senior undergraduate students at the school. Because the intervention was delivered within a course, it was not possible to assign participants randomly to the intervention and control groups, but we did eliminate the risk of selection bias through broad recruitment and inclusivity.

Our study protocol was approved by the Conjoint Faculties Research Ethics Board at the University of Calgary (REB17-2145).

### Participants

There were 28 participants in the full study. Fifteen were in the intervention group (IG), though one did not complete the quantitative tests, and 13 were in the control group (CG). See Table 1 for basic demographic information.

**TABLE 1:** Demographic information by group.

	Intervention group (*n* = 15)	Control group (*n* = 13)
Gender	60% female (0.7% undisclosed)	77% female
Age	20–40, *M* = 24.12, *SD* = 5.50	19–30, *M* = 23.23, *SD* = 3.53
Mental health diagnosis reported by student (anxiety, depression, and ADHD)	8	5
Academic discipline	6 drama, 4 music, and 5 dance students	6 drama, 4 music, 3 dance students
Improvisation experience	5 students with 4+ years of experience	4 students with 4+ years of experience

### Intervention

The intervention was delivered in the form of an intensive course on PGS for 21 graduate and senior undergraduate performing arts students. Fifteen of these students participated in the study. The course had the educational aim of teaching how to perform and create PGS. The course was not altered for the purpose of the study. That said, the research team was aware of the cognitive demands involved in the taught praxis and the potential effects on EF that such demands might produce. The course took place over eight continuous weeks in studio spaces. During the first 5 days, the group worked for 7.5 h per day. Each day, a new PGS was introduced through articles on and archival recordings of the artistic approach, a mini-lecture on both the approach and key concepts associated with it, group discussions, practical exercises, and practical rehearsal. One third of each day was dedicated to analytical work and two thirds were spent on experiential components. Participants were tasked to upload a written reflection to an online learning system each evening. On day 1, the group was taught a dance system called “Futuring Memory.” It was created by the Canadian choreographer Ame Henderson and the company Public Recordings for the 2013 work *relay* (Forté and Zimmer, 2011; Hansen, 2014, 2015). On day 2, the American 1960s avant-garde composer Cornelius Cardew’s indeterminate scores were introduced. The group learned “Paragraph 7” for vocal performance from *The Great Learning* (Cardew, 1971). On the third day, the group’s attention was turned to the Canadian theater director Paul Bettis’ theatrical “rule plays,” specifically *The Freud Project* from 1996 (Bettis, 2004; Hansen, 2008). The fourth day, the group returned to dance systems with a focus on the Canadian choreographer Christopher House’s version of the American avant-garde choreographer Deborah Hay’s solo commissioning scores and praxis (Hay, 2000, 2007, and 2013; Hansen and House, 2015). The fifth day was dedicated to the participants’ own creation of PGS. After this intensive week, participants were tasked to practice performing one or several of the taught systems for at least 45 min per day, 4 days per week. Their practice was restricted to the taught systems for the first 3 weeks. From the fourth week, participants were given the option of adapting the systems to address their needs as performers. Participants were required to upload a reflection post at the end of each of the seven practice weeks.

The analytical reflection on key concepts was designed to help students identify the procedures of each system and arrive at a preliminary understanding of how they generate performance. Experiential knowledge was then built onto this understanding, rendering it operational. This choice was based on the results of a pilot study into embodied and conceptual learning through PGS that Robert Oxoby and Pil Hansen completed in 2016 (Hansen and Oxoby, 2017). In this pilot, qualitative data revealed that students who engaged with both the conceptual analysis of the systems and participated in the experiential exploration of them advanced beyond students who only engaged with one of these components. Just like structural analysis has proven essential for the advancement of music performance (Bordin, 2017), we found that conceptual analysis of how a system is designed and works is relevant for PGS students’ advancement.

The four systems in the intervention share the characteristics described in the theory section, and thus they likely place a series of comparable executive demands on the performers. What follows is a detailed practical and procedural introduction to these specific systems that accounts for the intervention focus.

In Henderson’s dance system, the performers’ memories of movement from past choreographies or everyday routines serve as sources. Performers pre-select these memories and practice recalling them. The system has three tasks: futuring, recalling memory, and futuring memory. When futuring, each performer is tasked to register their co-dancers’ movement, form a hypothesis of what their movement will be in the next moment, and perform it. The objective is to reach unison (synchronized movement) collectively. The primary rule of this system is: do not copy. If an association arises to a source memory while futuring, then the performer must recall the memory and the group is tasked to future the recalling performer. The recalling performer allows the memory to be influenced by the futuring proposals. The generated choreography varies depending on how many source memories the group has available and how many different styles of dance the performers are trained in. Exhaustion is also a significant factor as the futuring task is demanding to sustain (Hansen, 2015).

To perform the music system, participants were given a printed score with a list of single words. A number and legend preceding each word indicates how many times and how loudly the word must be repeated. The system task unfolds as follows: performers begin by each selecting a pitch and a position in space. When prompted visually, they start singing the first word on the list in their selected pitch. After singing the indicated number of repetitions, each performer is free to move in space and tasked to seek out a new pitch from another performer. When they have found such a pitch, they once again position themselves in space and begin singing the second word in this pitch. The work continues this way until everyone has sung the full list of words. The score and the pitches comprise the sources of this system. The rules are simple and govern when each performer can move in space as well as the fact that they are not permitted to add new pitches or words to the system. The generated composition shifts depending on variables such as participants’ choices of pitch, tempo, pronunciation, sonic emphasis, position in space, vocal stamina, breath, spatial reverberations, and mistakes (Bertolani, 2018).

In preparation for the theater system, performers select source texts (plays, poems, theses, etc.) on a theme and devise transitional sentences. The latter are typically polite phrases that can be used to enter or exit a space, ask for permission to touch, or voice an opinion. The system the group worked on was devised on the theme of Freudian sexual transgression and gender/power roles. Performers added their own texts to sources first selected by Paul Bettis and his ensemble. These texts were hidden in properties and furniture within the rehearsal space, while transitional sentences were memorized. In pairs of three, participants started their system tasks by drawing cards that determine their character (mother, analyst/father, or daughter), their space (living room, office, or garden), and their objective (seduce, be seduced, observe). The style of performance of this rule play is exaggerated, melodramatic, and non-realistic. Rules include asking for consent before touching or entering a space, only using the selected text for improvisation (no added words), and responding to tasks prompted by light or sound cues. An outside manipulator is playing music and using spotlights and sound makers, which respectively prompt performers to dance a waltz, create a family tableau, read facts about Freud, perform a monolog, or hurry up and complete the character objective within 2 min. The generated story of this system varies depending on the texts, props, and space used and the performers’ ability to mobilize them during interactive scenes. The actors’ ability to transition when a new task is prompted and their competitiveness are also significant variables (Hansen, 2008).

In Hay and House’s dance system, the source is comprised of a score with concrete and abstract tasks like “open up the space like a fan,” or “three steps forward, two steps back.” Performers memorize these tasks, but interpret them and respond to them differently each time they perform them. While performing this score, each participant is tasked to sustain Hay’s practice. The foundation of this practice is a requirement to continuously draw in information from one’s surroundings and body, respond to it, let it go before an idea takes shape, shift the point of attention, draw in new information, and so on. Rules include not repeating oneself, not planning, rejecting learned responses, and rejecting sequencing. The generated choreography varies depending on how performers interpret the score tasks, their ability to inhibit planning and repeatable or trained responses, and their ability to sustain continuous attention shifting (Hansen and House, 2015).

All four systems require performers to draw on pre-selected sources for performance generation (i.e., memories of choreography, memorized musical or choreographic score components, or a repertoire of text-based lines). While recalling these sources, the performers are inhibiting trained movement and vocal responses, other memories of choreography, or improvised impulses. The systems also require performers to draw on new information selectively while responding to it physically, vocally, or in verbal and physical actions within a set of restrictive rules. Each system explicitly directs the performers’ attention to different types of sensory stimuli: the dance systems mostly rely on visual and proprioceptive information, the music system primarily draws on auditory information, while the drama system emphasizes visual and auditory information. Both dance systems require continuous attention and set-shifting, while the music and theater systems alternate between a singular focus of attention and the need to rapidly shift attention and set when seeking out a new source or transitioning to a new task. The complexity of the tasks and rules also differ. The music system is the simplest, as its tasks are sequential instead of simultaneous and the rules remain constant. The dance systems involve three simultaneous tasks with some variation of both rules and tasks over the course of the performance. The theater system includes three simultaneous tasks at all times, but one of them changes sequentially, and the rules are complex. While some familiarity with the source materials, tasks, and rules is useful and helps performers achieve fluidity of performance, too much familiarity tends to lead to predictable repetition that stops the system from generating new performance.

### Quantitative Test Instruments

The Delis–Kaplan Executive Function System (Delis et al., 2001) design fluency tests were administered to measure the higher-level executive functions of inhibition and flexibility. Participants were asked to create novel designs by drawing lines between dots within a set time limit, under three increasingly more complex conditions. The first condition asked participants to connect dots on the page. The second condition required participants to only connect certain dots and ignore incorrect dots. Finally, the third condition required participants to switch between rules for connecting dots (e.g., “connect dots of alternating colors”). Condition 1 is designed to test the higher-level executive functions of fluidity and problem-solving initiation, condition 2 measures inhibition in addition to these functions, and condition 3 furthermore tests flexibility. Conditions 1–2 also measure the more basic cognitive skills of visual attention, motor speed, visual perception, and simultaneous processing. Composite scores are also created. These tests were administered to the intervention group within 10 days before the intervention and within 2 days after the intervention. The control group was tested within the same time interval. In compliance with the standard D-KEFS procedure, age-based norms were used to determine the scaled scores of correct designs under each condition as well as the composite scores across all conditions.

### Qualitative Data Collection and Processing

The intervention group was tasked to upload daily reflection postings throughout the first intensive week and weekly postings throughout the seven practice weeks. In each posting, they were instructed to include at least four observations on how the system works and how it affects them as performers that could be based on any of the materials or experiences from the day or week. All reflection postings were transferred to the qualitative coding software NVIVO as participant-specific cases.

These data sets varied in posting volume and frequency, depending on how much participants wrote and whether they uploaded all or only some of the requested postings. Three participants who uploaded less than 2/3 of the required posts were not included in the analysis, as findings otherwise might reflect their limited data rather than their self-reported experiences and reflections. This elimination affected the distribution within the intervention group of gender (10F, 1M, 1 non-disclosed), discipline (five drama, two music, and five dance students), and significant improvisation experience (IG 4 with 4+ years of practice). The rest of the variables were unchanged. Although the volume (word count) of writing uploaded by the remaining 12 participants varied (range: 3,300–10,200 words, median: 7,800 words), this difference was found to mostly reflect whether each participant was reporting their observations directly or prefacing each observation with sections describing experiences in detail. This difference was addressed by coding observations, not details.

The data sets were coded to saturation using an inductive, grounded theory method. The work progressed from identification of concepts (open coding), through comparative analysis and discovery of relationships (generation of categories and axial coding), to analysis of these relationships and generation of theory (selective and theoretical coding). In particular, a word frequency search was first completed to identify concepts used by multiple participants to reflect on their praxis. More specified word searches were then completed on the terminology of these concepts and the results were auto-coded as possible categories. Returning to the case materials, the coded contents were reviewed for accuracy, text that did not belong in a category or derived from descriptive details was un-coded, and emerging sub-categories were coded at subcodes. To begin identifying possible relationships between the generated thematic categories, a cluster analysis was visualized, identifying codes with related and comparable coding patterns. A more detailed comparative analysis was completed of such clustered code contents to identify actual relationships. In the final phase, case materials were revisited with selective, but more detailed, attention to coded contents that contribute to the identified relationship and theory was articulated.

## Results

### Cognitive Effects

The sample for our quantitative analyses included 13 participants in the control group and 14 participants in the intervention group. Given the small sample sizes and sample heterogeneity inherent in this type of research, data was analyzed using non-parametric Wilcoxon tests, which impose no assumptions on the distribution of the underlying data. Rather, this test looks at the distributions of the control and intervention populations and takes as the null hypothesis that the two populations are drawn from the same sample. This technique was used for both combined and individual Design Fluency (DF) test items. Wilcoxon tests provide a lower-bound on the significance of our results. We further explored the data from the experiment by conducting one-tailed *t*-tests to identify results in which the Wilcoxon tests were indicative of changes due to the intervention.

**FIGURE 1:**
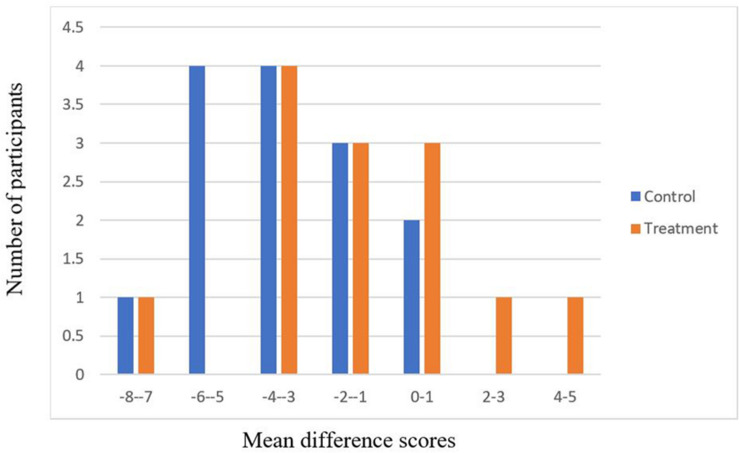
Scaled DF tests’ distribution of pre- and post-test differences for condition 2.

Looking at the initial distribution of DF scores, we cannot identify any differences between the pre- and post-intervention tests administered to participants across the control and intervention groups. As such, we chose to look at the data using a difference-in-difference approach in which we compare the changes (positive scores indicating improvement) across the pre- and post-administered tests to participants. This yields distributions of “post-score minus pre-score” for the control and intervention groups. The non-parametric tests identify an intervention effect with respect to the scaled scores in condition 2 of the DF tests [Wilcoxon *p* = 0.054; *t*(26) = 4.2, *p* = 0.02]. This result suggests that the intervention increased performance over the pre- and post-test period. Figure 1 presents the distributions for this scaled test.

Thus, our quantitative data suggests a marked change in the pre- and post-test performance of subjects on the DF tests. The key to notice in these distributions is the upper tail observed in the intervention group, suggesting that the intervention resulted in improved performance. This result is bolstered by the results of scaled scores in conditions 1 and 3. Although we fail to identify significant differences at the 5% level for conditions 1 and 3, we do see that Wilcoxon tests reject the null hypothesis at the 10% level for these conditions. This difference may in part explain a trending effect we found on the composite, scaled DF test score [Wilcoxon *p* = 0.0696; *t*(25) = −2.06, *p* = 0.025].

Although outliers and our small sample size reduce the statistical power of the sample, we do identify changes occurring as a result of the intervention, thereby illustrating the potential for our intervention (and PGS more generally) to yield changes (here improvements) in test performance.

### Experiences of Mediating Processes

**FIGURE 2:**
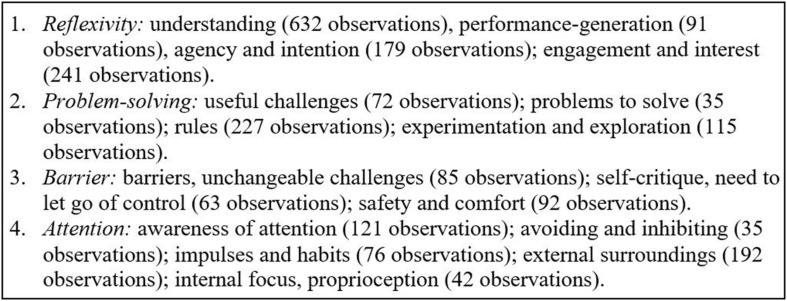
Clusters of related primary categories.

Our analysis of qualitative data from the mediating process revealed behavioral patterns. From these patterns, we learned how the demands of the intervention practice were experienced by the participants and how they responded. These insights provide additional explanations of the measured effects and raise questions of relevance to future research and implementation that will be addressed in the discussion and implications sections of this article. In this section, we first explore relationships between the primary categories of participant observation that our coding process revealed (Figure 2); then we offer analytical theory inferred from these relationships.

All participants *reflected* frequently on their own thinking process and on their analytical and conceptual understanding of the systems as both evolved throughout the intervention. It is therefore evident that our choice of complementing experiential learning with analytical reflection in the first five intervention days ended up informing the participants’ independent practice weeks. Reflections posted in cluster 1 were primarily centered on how the system can continue to *generate performance* and the performer’s *agency* to make choices within the system. These analyses often contributed to thinking through factors when addressing a challenge, developing ideas, or planning the next phase of practice. Reflections on *engagement, interest*, and *agency* remained high through the intensive days and the first three practice weeks, they dropped significantly in weeks 4 and 5, and resurged in week 6. Note that week 4 was when participants were given the option of adapting the systems in order to ensure that they continued to generate performance and address their needs as performers.

This delayed positive effect on *engagement, interest*, and *agency* can be explained by the participants’ experiences of *barriers* and *challenges* (clusters 2 and 3). In the first weeks of working independently on the practice of the systems, the quantity of observations on challenges that were experienced as respectively unchangeable and useful grew. In week 2, individuals who reported unchangeable challenges began to describe them as barriers; and in the third week, reports of useful challenges dropped altogether while the experience of unchangeable challenges and barriers surged. Looking at the contents of the postings, the tendency reflects a learning curve. The more ‘skilled’ participants became at performing the systems, the harder it was for them to avoid repeating themselves and find fresh responses while performing. They reported feeling stuck with some of the tasks and becoming predictable in performance. In week 4, when participants were given the option of adapting the system, the pattern changed radically: challenges were predominantly reported as useful experiences again and reflections on agency shifted from a focus on choice-making to the participants’ work on manipulating system rules. This development was sustained through the intervention, but it was not until week 6 that reports of unchangeable challenges and barriers dropped altogether. Looking more closely at the experiences that participants shared after week 4, many of the first ideas for adaptation failed because they introduced rules that were too restrictive. It took several attempts before more simple solutions, such as adding objects to a space, interpreting a task differently, or redirecting the performer’s attention were tried out systematically instead. Those solutions reflected a stronger understanding of how the systems generate performance, they were experienced as more useful in practice, and they led to a surge in reports of engagement and agency.

As indicated above, participants approached challenges with distinct problem-solving or barrier attitudes. Each of these attitudes were associated with different behavioral patterns. The first manifested in observations of challenges that were regarded as *useful experiences* leading to new realizations or as *problems to be solved* strategically.

I think that the magic of this system lays within its problem-solving component, and the harder this problem is, the more interesting it is to watch. Although, a choreographer could of course implement layers of challenges (intervention group participant).

Data coded at these categories are closely related to discussions of *rules*, including attempts to adapt them, and a tendency to frame the work as *experiments* or *explorations*. When this problem-solving attitude was expressed, challenges were perceived as changeable situations and approached with experimental attempts to manipulate rules and explore system options in search of solutions.

Some problems that came up with my score include: transitions, inconsistent duration of each phase, wanting to be more internal than external, not having an actual audience, and wanting a bigger challenge.

Possible solutions: create a task specifically for transitions, allow myself to be inconsistent with the duration of each phase or set up an alarm, be strict with the duration and task of each phase to avoid being more internal than external or the other way around, invite someone to be my audience, think about a bigger challenge (not being allowed to move the same body part twice in a row? Not being allowed to use the same movement vocabulary? To play with rhythm? To sing a song or speak while performing the score? Tell the audience a story?) (intervention group participant).

This reaction required analytical meta-reflection on system components and how they affect the performer rather than impulsive responses.

The other distinct attitude is evident in observations that represent challenges as *unchangeable* situations or *barriers* to the participants’ performance. These categories were often related to comments on establishing a *safe* space, on feeling *safe* working with others, and on returning to a familiar source or task because it makes a participant feel more *comfortable*.

I liked being in my safe zone of blurry dance rather than pushing beyond into more of the score. Despite my struggles to leave my comfort zone, I made it happen closer to the end and sang the song. Even though I struggled with the praxis overall, it felt like a very good day (intervention group participant).

For a subgroup of participants who reported anxiety, the barrier attitude was also closely associated with *self-critique* and reflections on the *need to let go* of control.

I struggled yesterday with where Futuring ended and Hay began … I found myself wanting to combine the two practices and struggled with it. When I let go and let the influence of Hay’s practice be part of how I worked, it became much easier to future (intervention group participant).

Most participants underwent a learning curve in which the barrier attitude grew from the second practice week and was replaced with the problem-solving attitude in the fourth practice week. Over the full intervention, this development meant that the two attitudes ended up being balanced. Two subgroups differed, though: a participant who reported having an attention deficit disorder, but no anxiety, leaned exclusively toward the problem-solving attitude from the outset and participants who reported anxiety leaned strongly toward the barrier attitude throughout the intervention.

Observations on *attention and awareness* are distributed evenly across most study participants with a range of 9–19 observations per person. The exception is two participants who reported having an attention deficit disorder; they only mentioned attention or awareness 2–3 times each in their postings. Interestingly, the participants with the highest amount of observations on attention all reported anxiety as well. In the category of *attention and awareness*, participants predominantly observed what they were attending to or how they were attending during performance. Focus on learned habits and the need to inhibit or avoid them is directly associated with the degree to which participants also discussed attention: participants with few observations on attention also shared few or no observations on the topics of habits and inhibition.

Participants with a high degree of reflexivity about *attention* did all report strategic choices of redirecting their attention toward new *external* or *internal* sources of stimuli or shifting their mode of attending. The latter did, for example, bring about a shift from focused to peripheral vision, from isolating a sound to listening more broadly, and from internal to external stimuli. Looking more closely at these strategies, they often overlapped with reports of experiencing unchangeable challenges or barriers. Instead of solving or removing the challenge, participants with strong reflexivity about attention worked around the challenge by redirecting their attention, taking in new sources of information, and responding differently while performing.

Because I was tired and I was by myself, I was very tempted to stop the system and do something else. The times [when] I felt I had to stop the system and do something else served as a tool to shift my attention to the space … I had fewer thoughts on where my body should go next, and I kept going and finding new exciting unexpected movements, as a reaction to my physical surroundings (intervention group participant).

Regardless of the degree to which participants were reflexive about attention, they generally described perceptual stimuli and discussed their experience of responding to them with performance. They were thus hyperaware of the need to take in new information and respond to it within the systems. In these descriptions, participants referred to their *external surroundings* (the space, sound, objects, other performers) to a much higher degree than *internal, proprioceptive* experiences. The external focus was typically used to draw new inspiration and the internal focus was commonly used to address feeling overwhelmed and needing to reduce the information used in the improvisation. Note that unlike engagement and enjoyment, which were significantly reduced during weeks 4 and 5, observations on *attention and awareness* remained consistent. A gradual decrease in the otherwise high number of observations on the external environment was related to a gradual increase in reports of internal perceptions, demonstrating the strategy of redirecting attention in effect.

## Discussion

### Quantitative Results

The quantitative results from the DF tasks on the D-KEFS support the finding that involvement in the PGS increased fluidity, inhibition, and problem-solving initiation. The results further indicate improved flexibility in thinking over the course of the intervention. Given our directional hypotheses, one-tailed tests were carried out to determine the change in EF skills. In particular, condition 2 of the DF task, where participants are required to inhibit a prepotent rule when creating designs, demonstrated a significant and consistent pre-post change, whereby there was greater improvement in fluidity, inhibition, and problem-solving initiation for those in the PGS intervention over those in the control group. Additionally, the composite scores of DF conditions 1–3, which also include tasks of shifting between rules, demonstrated a trending pre-post change, which indicated that the PGS intervention group experienced greater flexibility in thinking than the control group. It is possible that the PGS techniques of creating novel responses, restricting or inhibiting previously demonstrated movements, and responding to new information helped to increase (or bring increased awareness to) participants’ fluidity, inhibition, problem-solving initiation, and flexibility in thinking, benefiting them on the DF task.

### Qualitative Results

The qualitative results offer analytical insight into the demands that PGS place on performers. Our study participants underwent a challenging learning curve because a more trained performance within these systems resulted in repeatable responses (prepotent, implicit memory). These responses worked against the tasks of inhibiting learned responses, taking in new sources of information, and producing fresh responses with awareness while performing that PGS generally involve. As performers, participants were attracted to the “comfort zones” of learned responses and began to experience score sections or tasks that felt less familiar as barriers. In addition to the inherent challenge of multitasking, which all the systems entail, the meta-challenge of addressing such barriers required the development of problem-solving and strategies for redirecting attention.

These strategies were arrived at through multiple cycles of reflexivity about experiences from the practice, analytical reflection about patterns of response, development of solutions, operational experimentation with them, new reflexivity about experiences, and so on. In other words, the demands on executive functions of PGS were not limited to the working memory span of the performance present. It was extended into the conscious development of operational strategies over longer durations and through cyclical exchange between high-level processing of problems and fully embodied experimentation with solutions.

These findings confirm and deepen our understanding of the benefits of conscious analysis, strategic reflection, and problem-solving in the context of improvisation practices (Hansen and House, 2015; Hansen and Oxoby, 2017). The results furthermore support the critique of an over-emphasis on presence in classical improvisation approaches, voiced by contemporary improvisors and PGS creators (Drinko, 2013; Sarco-Thomas, 2014; Midgelow, 2015; Hansen, 2018). Without the PGS-specific tasks and rules that restrict responses, performers would have the option of staying within their comfort zones and repeating responses already arrived at. PGS clearly drive performers to become aware of such tendencies and to counter them by adding new tasks, rules, and strategies that produce novel performance. In PGS, performance fluency is not reflected in repeatability, but rather in the ability to apply a complex and changing set of rules and attention-shifting/problem-solving strategies during continuous performance.

### Integrated Perspective on Results

In summary, the quantitative results support the theory that PGS have a significant effect on specific EFs, whereas the qualitative results help us understand why. The participants’ continuous qualitative observation of external perceptions, which registered while working on physical tasks, indicates that the practice placed high demands on basic cognitive skills measured by the D-KEFS: visual attention, visual perception, motor speed, and simultaneous processing. This indication supports the finding of a strong effect in condition 2 of the DF Test. The need to not only advance through implicit learning and fluid performance of multitasking skills, but also to remain reflexive about how those skills affect the performance and develop strategies to refresh responses, placed an additional layer of demands on executive functions.

Combined, the strategies participants developed effectively ensured that they continued to rely on inhibition (blocking learned responses), initiation of problem-solving (reflecting on challenges and adapting the system), and flexibility (redirecting attention in search of new responses, shifting between modes of attention, shifting between rules) while multitasking with fluency during performance. These demands explain the respectively strong and trending effects measured on higher-level executive capacities in condition 2 and in the composite score of the DF test.

### Limitations

As with any intervention research, a number of limitations are noted. In particular, it is noted that the sample size is small, therefore limiting the number and type of quantitative analyses that could be carried out. As well, one participant did not complete the quantitative measures, resulting in an uneven sample size. There was heterogeneity within both the control and intervention groups (e.g., presence of mental health or attentional concerns) that could not be further explored given the sample size; it is possible that the presence (or absence) of a mental health condition could impact results in this type of intervention.

It should also be noted that the smaller sample size may limit the generalizability of these findings. However, the artistic practice of PGS cannot be taught to larger groups as the integrity of the practice may be compromised. As such, while the current sample size is small, it allowed for a detailed overview of the intervention. This overview reflects the short-term effects of the intervention as long-term effects have not been measured.

### Implications

The results indicate that simple PGS interventions designed for different populations could potentially help strengthen and maintain EF abilities over and beyond performing arts interventions focusing on structured or memorized material (e.g., Meng et al., 2019). It is also possible that introduction of PGS components to simple or classical improvisational interventions (such as the interventions of Karakelle, 2009; Thaut et al., 2009; Coubard et al., 2011; Biasutti and Mangiacotti, 2018) can increase their effect on key EFs. However, further studies are needed to determine range of transferability.

Understanding the EF demands of PGS can help creators and teachers of these systems calibrate tasks and rules to their performers’ problem-solving and set-shifting abilities, ensuring that the systems neither become so easy that performers begin to repeat themselves nor so difficult that barriers become insurmountable. Insight into the barriers, problem-solving, and strategies involved in performers’ learning curve as they practice can also be beneficial when developing systems and planning rehearsals.

The combined quantitative and qualitative results of this study provide evidence of the higher-level cognition involved in PGS and how important it is for the performers’ learning curves. These findings can be extended to other forms of improvisation that are complex and structured and thus likely to involve set-shifting and inhibition. The results may encourage teachers of such approaches to reevaluate the discourse of presence and teaching methods that aim to suppress reflexivity, problem-solving, and strategy.

The combined findings also point to the importance of closely considering the specific cognitive demands of performing arts interventions. This is both relevant when studying the cognitive effect of such praxes and when aiming to apply them for educational or therapeutic purposes. EFs are not typically affected when interventions involve imitated and structured performance for memorization and recall, and different EFs are affected depending on whether interventions include simple improvisation tasks, open forms of improvised creation, or more complex and demanding improvisation tasks. By taking such considerations one step further and analyzing participants’ behavior during the interventions as mediating processes, the cognitive demands of interventions and the behaviors they generate are better understood. It is this more interdisciplinary and integrated level of insight that renders findings applicable to both psychology and the performing arts.

To further investigate or apply our findings we make the following recommendations:

1. *Examination of interactions between mental health and PGS-demands:* Complete a detailed qualitative case study to better understand interactions between mental health and the PGS-demands that we now know affect executive functions.
2. *Development and determination of scientific/therapeutical transferability:* Integrate PGS features in improvisation interventions, which can be delivered to larger groups, and test whether they result in comparable short-term effect as well as long-term effects on higher-level cognition across different population groups.
3. *Application to performing arts education:* Integrate reflexivity, problem-solving, and strategy discourse and tasks in the teaching of complex improvisation in the performing arts to support the students’ learning curve.
4. *Developmental application to professional performing arts praxis:* Complete Practice-as-Research experiments with the development of PGS, matching levels of cognitive demands, such as task complexity and energy sources, to the performers’ capacity and gradually increasing these levels.

## Data Availability Statement

The datasets presented in this article are not readily available because the data is confidential, as required by our ethics protocol.

## Ethics Statement

The studies involving human participants were reviewed and approved by Conjoint Faculties Research Ethics Board, University of Calgary (REB17-2145). The patients/participants provided their written informed consent to participate in this study.

## Author Contributions

PH led the study and intervention, performed the qualitative analysis, and wrote the first draft of the manuscript. PH and EC completed the literature review, performed the cross-disciplinary analysis, and contributed to manuscript revision. RO performed the statistical analysis. EC and RO wrote sections of the manuscript. All authors contributed to the conception and design of the study and read and approved the submitted version.

## Conflict of Interest

The authors declare that the research was conducted in the absence of any commercial or financial relationships that could be construed as a potential conflict of interest.

## Footnotes

1 Meta-analyses of empirical studies into the cognitive effects of theater and music are not available. The field of theater psychology is emerging and features few evidence-based publications. The field of music psychology is significantly larger, but reviews focus on narrow population groups and areas of cognition (e.g., cross-cultural perception, dementia, and emotional control). There are no reviews available on executive functions and music, but reviews on music therapy from 2013 and 2017 with focus on dementia make brief mention on EF (McDermott et al., 2013; Fang et al., 2017). Unfortunately, the review from 2013 does not account for the kinds of intervention offered and the 2017 review does not mention improvisation. Both reviews conclude that the body of research available focusses on emotional adjustment over cognitive effects and is limited, leaving the area of music therapy and cognition with a significant gap.

2 The term ‘performance generating systems’ categorizes comparable approaches that creators refer to as dance machines, rule plays, praxis scores, and indeterminate scores.

3 Note that the study leader, Pil Hansen, was co-teaching this course. For ethical reasons, Hansen was thus not recruiting or testing participants and did not have access to the participants’ surveys and test data until after final course grades had been submitted and the appeals period was concluded.
